# Local and systemic immunological parameters associated with remission of asthma symptoms in children

**DOI:** 10.1186/1710-1492-8-16

**Published:** 2012-10-08

**Authors:** Susan Waserman, Parameswaran Nair, Denis Snider, Mary Conway, Lata Jayaram, Lynn M McCleary, Jerry Dolovich, Frederick E Hargreave, Jean S Marshall

**Affiliations:** 1 Departments of Medicine, McMaster University, Hamilton, ON, Canada; 2 Departments of Pathology, McMaster University, Hamilton, ON, Canada; 3 Departments of Pathology, Dalhousie University, Halifax, NS, Canada; 4 Microbiology & Immunology, Dalhousie University, Halifax, NS, Canada; 5 Department of Allergy and Clinical Immunology, McMaster University Medical Centre, 1200 Main Street West, Hamilton, ON, Canada

**Keywords:** Asthma in children, Eosinophils, IL-5, TNF-α, IL-12

## Abstract

The immunological and clinical parameters that are associated with asthma remission are poorly understood. The cytokine and local mediator changes associated with the resolution of asthma symptoms were examined in three groups of subjects 12–18 years of age (n = 15 in each group): (a) continuing asthma group (CA) who had persistent symptoms since early childhood, (b) an age, sex and atopic status-matched group who had persistent symptoms in early childhood but in whom these had resolved (RA), and (c) a non-atopic, non-asthmatic control group. Clinical parameters, sputum cell counts, peripheral blood mononuclear cell (PBMC) cytokine production and activation marker expression were determined. All of the CA had methacholine airway hyperresponsiveness compared with only half of the RA subjects. The CA showed elevated numbers of eosinophils and increased ECP and IL-5 in sputum, which were not observed in the RA. PBMC cytokine studies revealed increased production of the type 1 cytokines IL-12, IFN-γ and TNF-α in the CA group compared with the RA group, under a range of activation conditions, however, the production of IL-4 and IL-5 were unchanged. These findings suggest that decreased type 1 cytokine expression as well as decreased eosinophilic inflammation is associated with the resolution of asthma symptoms.

## Introduction

Asthma is characterized by episodic symptoms of wheeze, breathlessness or chest tightness due to variable airflow obstruction and increased airway responsiveness that may or may not be associated with bronchitis. The prevalence has been estimated at 10-20% in children [1,2]. Wheezing is more likely to persist and develop into asthma in children with atopy, a positive family history, (especially maternal) of asthma or atopy, airway hyperresponsiveness, and exposure to indoor allergens or cigarette smoke [3-11]. Wheezing associated with the development of asthma tends to begin at age 3 or persists beyond this age [12].

Indications of a predisposition to asthma are often present early in life, possibly even before birth [13-15]. Although atopy may be evident in early infancy, the generation of pulmonary responses appears to require local sensitization of the airways to aeroallergens, a process that may require allergen exposure for 2–3 years. By age 3, atopic children with recurrent wheezing illnesses exhibit increased mast cell and eosinophil numbers in lavage fluid [12]. Upon exposure to allergen, sensitive individuals will respond with rapid mast cell degranulation and activation of antigen presentation, and the recruitment of effector cells such as T cells and eosinophils. This inflammatory response may become chronic, if allergen exposure is not terminated or if appropriate treatment is not administered. Indeed, chronically activated memory T cells are observed in asthma [16], with an enhanced capacity to produce IL-4 and IFN-γ [17,18]. During this consolidation phase, structural damage due to persistent inflammation results in remodelling of the airways, leading to a degree of fixed airflow limitation and possibly greater hyperresponsiveness [19-24].

A proportion of children with asthma will “outgrow” their disease, and become symptom-free as adults, [5,7,25] even though many of them remain hyperresponsive [10]. A number of factors have been associated with the persistence of asthma into adulthood, including female gender, increased severity of symptoms in childhood, high serum IgE, low FEV1 or PC20, and those with perennial symptoms in response to house dust mite or molds [8-10,20]. While these correlations have been made, there is little understanding of the immunological mechanisms by which asthma develops into a persistent disease, or by which symptoms regress.

Expression of cytokines can profoundly influence immunological processes. Allergic diseases are critically dependent upon the development of Th2 cells, especially as a source of IL-4 which is critical for IgE antibody production. T-helper cell differentiation is influenced by the strength of T-cell receptor MHC interaction and the nature of co-stimulation, but most importantly by the presence of IL-12, which promotes Th1 differentiation, IL-4, which promotes Th2 differentiation, and IFN-γ, which tends to antagonize the development of Th2 cells. Activated Th2 cells are prolific sources of IL-4, IL-5, and IL-13, among other cytokines, each of which has been suggested to play a role in asthma pathogenesis. Peripheral blood mononuclear cells (PBMCs) from asthmatic and atopic patients have been shown to produce Th2-associated cytokines upon stimulation with allergen, [17,26-29], non-specific stimuli such as PMA or anti-CD3 [27,28,30] and in the absence of experimental stimulation [31]. Type 1 cytokines such as TNF-α, IL-12 and IFN-γ have a pivotal role in enhancing the infiltration and activation of effector cells in host defence and many inflammatory diseases, and may also play an important role in asthma pathogenesis.

The objective of this study was to investigate the clinical and immunological parameters which may be associated with resolution of asthma symptoms. Adolescent subjects with continuous asthma, those in whom childhood asthma symptoms had resolved and non-atopic controls were compared with respect to a number of criteria including: inflammatory cell content of the sputum, cytokines in sputum and cytokine production by peripheral blood mononuclear cells (PBMC) and the proportions of peripheral blood T cell subsets and activation markers.

## Methods

### Subject selection

Three groups of subjects were examined in parallel triplets (one from each group). Each triplet group was matched for age (within 1 year) and sex. Since growing out of asthma is reported to be more likely in children with mild or no allergy we endeavoured to match group 1 and 2 for similar levels of positive skin tests using a panel of 14 common inhalant allergens.

#### Resolution of asthma symptoms group (RA)

Fifteen children were recruited who had a history of at least one year of persistent symptoms consistent with asthma between the ages of 2–6 years inclusive who were now aged between 12–18 years inclusive, and had been completely and continuously free of asthma symptoms for at least one year. They were identified by questionnaire/ interview and examination of medical records. Persistent asthma at ages 2–6 was necessarily defined based on symptoms (ie. cough, wheeze, breathlessness) considered to need asthma treatment including inhaled/oral corticosteroids, inhaled/oral bronchodilators, theophyllines, or cromolyn . The absence of asthma symptoms between the ages of 12–18 years was determined from a questionnaire which included no asthma symptoms during vigorous exercise or respiratory infection, and no need for asthma medication during the past year. A cough was permitted provided it was limited to throat clearing or less than five coughs per day in the last month.

#### Continuous asthma group (CA)

Fifteen children aged 12–18 years inclusive were recruited with at least one year of persistent symptoms consistent with asthma between the ages of 2–6 inclusive and who now had persistent asthma. Persistent asthma (ages 2–6) was defined in the same way as for the RA. Its continued presence was confirmed by airway hyperresponsiveness to methacholine (PC20 < 8 mg) or an improvement in FEV_1_ ≥ 15% from baseline measurement 15 minutes after receiving 200 μg of Salbutamol in addition to daily symptoms and the need for asthma medication.

#### Non atopic asymptomatic group (Control)

Fifteen subjects ages 12–18 who had no past or current history of allergy or excessive respiratory symptoms and who had completely negative allergy skin tests.

All subjects were required to have an FEV1 of > 70% predicted. None of the subjects had eczema, active rhinitis requiring nasal steroid therapy, were past or current smokers, were pregnant, had a chest infection within the past month or had received allergen immunotherapy. All study assessments were made outside the seasonal allergy season, and children were instructed to try and keep their perennial allergen exposure constant through the assessment period. The study was approved by the St Josephs Healthcare Research Ethics Board and written informed consent was obtained from both subjects and parent/guardian.

### Study design

This was a cross sectional study with two visits within 3 weeks. In visit 1, all subjects completed clinical questionnaires, provided informed consent, underwent a physical examination and had allergy skin tests. At this time, subjects also performed a methacholine inhalation test, had a urine test for pregnancy (where applicable) and subjects in the CA group were provided with salmeterol and salbutamol. At visit 2, spirometry was performed including bronchodilator reversibility, sputum and blood samples were taken for multiple analyses (see below). Remaining salmeterol and salbutamol were collected from subjects in the CA group.

Inhaled corticosteroid medication was discontinued in the CA group 2 weeks prior to subject testing and sample collection. At the time inhaled steroid was discontinued, 2 puffs bid salmeterol was provided. Forty eight hours before samples were taken, salmeterol was stopped and substituted with salbutamol 2 inhalations qid. Salbutamol was stopped at least 6 hours before blood samples were taken. Laboratory testing was performed simultaneously on three (matched) subjects, one from each group. Laboratory personnel performing the tests were not informed of the groups to which each subject belonged.

### Procedures

Routine physical examination, questionnaire and clinical tests were performed according to standard protocols. Spirometry included measurement of FVC, FEV_1_, FEV_1_/VC and methacholine challenge tests. Blood counts included a manual eosinophil count.

### Sputum cell induction and examination

Sputum was induced with an aerosol of inhaled hypertonic saline by a modification of the method of Pin *et al.*[32] after pretreatment with inhaled Salbutamol 200 μg. The modification consisted of inhaling the hypertonic saline in concentrations of 3, 4 and 5% each for 7 minutes. Sputum examination followed the method of Pizzichini *et al.*[33]. Sputum mediator assessments were performed using routine protocols. The concentration of eosinophil cationic protein (ECP) was determined using a sensitive radioimmunoassay (Kabi-Pharmacia Diagnostics AB, Uppsala, Sweden). Fibrinogen was measured using an “in house” ELISA assay which employed rabbit anti-fibrinogen antibody (Dako# A080). Cytokines were assayed by ELISA (IL-5 using Biotrak kit, Amersham, UK and TNF-α by “in house” ELISA).

### Peripheral blood cytokine analysis

Peripheral blood mononuclear cells (PBMC) were isolated from the blood of each subject. Matched triplets which included one subject from each group were sampled and assayed in parallel. Short term cultures of PBMC were established for 24 or 72 hours in the presence or absence of a series of stimulants. These included anti-CD3 antibody 1 μg/ml (OKT3, Cedarlane , Burlington, Ont), killed *Staphylococcus aureus* Cowan strain 1 bacteria (SAC) 0.07% w/v (Sigma, St Louis, Mo). *E-coli* derived LPS (Sigma) 5 μg/ml, Tetanus toxoid (0.1 μg/ml) and a combination of phorbol ester PDBu (Sigma)(10^-6^ M) and Calcium ionophore A23187 (Sigma) (10^-7^ M). The concentrations and timing of these stimuli were selected on the basis of pilot experiments.

The cytokine content of cell free supernatants, was assessed using ELISA kits with the exception of TNF-α which was assessed using a well validated “in house” ELISA. The cytokine kits employed were: IL-4- Biotrak RPN 2753, IL-5- Biotrak RPN2761, IL-6 Biotrak RPN 2754, IL-10 Biotrak RPN 2755, IL-12- Biotrak RPN 2765, GM-CSF Biotrak RPN 2757. All of these kits were obtained from Amersham (Toronto, ON, Canada).

### Flow cytometric protocols and analysis

**Figure 1:**
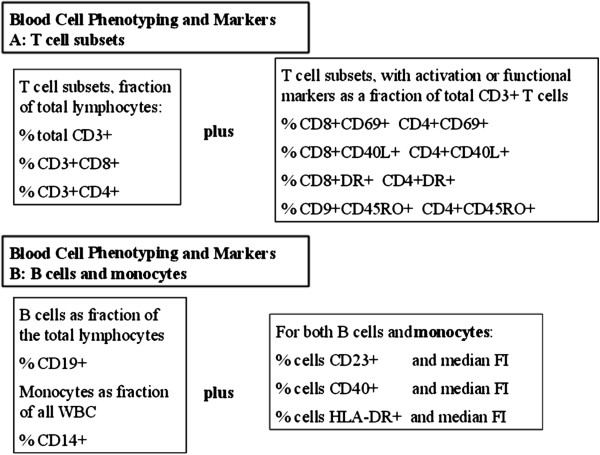
**Blood Cell Phenotypes defined for analysis of flow cytometric data.** Major lymphocyte subsets are reported as % of total lymphocytes. Activated or functional lymphocyte subsets are reported as % of each lymphocyte type. Expression of functional B or monocyte surface markers are reported as median fluorescence intensity.

Panels of antibodies were employed in 2 and 3 colour analysis protocols to identify and calculate frequencies of T-cell subsets, B cells, monocytes and subsets of these cells expressing important functional markers. A complete listing of B and T lymphocyte, and monocyte phenotypes analysed by flow cytometry are listed in Figure 1. All staining was done in whole blood with subsequent fixation and wash. Flourescent labelled antibodies against CD3, CD4, CD8, CD14, HLADR, CD45RO CD45RA, CD40 and CD23 were purchased from Becton Dickinson Canada (Mississuaga., Ontario). Antibody to CD40 ligand (CD40L) was purchased from Beckman-Coulter Canada (Mississuaga, Ont).

### Statistical analysis

Non parametric statistics were used to test for differences between the subject groups. Two sets of analyses were conducted, one testing for differences among the three subject groups (RA vs CA vs control) and one testing for diffrences between the two asthma groups ( RA vs CA). Between group differences were for each parameter of interest with statistical significae for all tests set at p = 0.05. Three way comparisons were tested using the Friedman test, the non-parametric equivalent of repeated measures ANOVA. Two way comparisons were tested using the Wilcoxon signed Ranks test, the non-parametric equivalent of the paired t-test. These provided conservative tests of between subject group differences.

## Results

### Clinical evaluation and spirometry

The subjects had a median age of 15, they included 10 sets of three matched female subjects and 5 sets of three matched male subjects. All subjects were examined in visit 1 and 2 as originally scheduled and described above with all asthmatic subjects completing a full 2 weeks without inhaled corticosteroid therapy. There were no significant differences between the subject groups for CBC, differential blood counts (Table 1) or ESR (data not shown).

**Table 1:** Clinical characteristics

**Parameters**	**Controls**	**CA**	**RA**
**Age**	**14 (12–18)**	**14 (13–18)**	**15 (12–18)**
**Gender (males), n**	**10**	**11**	**11**
**FEV1**	**3.8 (2.2-5.2)**	**2.8 (1.9-3.8)**	**3.7 (2.1-5.1)**
**FEV1,%**	**101 (88–120)**	**85.1 (62–105)**	**99 (70–123)**
**FEV1/VC**	**94 (84–112)**	**69.4 (50–93)**	**82.5 (58–100)**
**FEV1 reversibility**	**4 (0–11)**	**11 (0–45)**	**3.8 (0–8)**
**PC20**	**16 (8–32)**	**0.85 (0.1-6.4)**	**9.1 (0.5-30)**
**Skin prick test**	**0 (by definition)**	**7 (3–14)**	**3 (0–11)**
Blood eosinophils,%*	**0.16 (0.05)**	**0.25 (0.07)**	**0.15 (0.04)**

Values are mean (minimum-maximum).

Median (minimum-maximum).

#### Methacholine challenge test

The CA subjects had a mean PC20 of 1.6 mg/ml. In contrast, control subjects had a mean PC20 of 17.09 mg/ml (all subjects had PC20 methacholine > 8 mg/ml). Eight out of fifteen RA subjects had a PC20 of less than 8 mg/ml while the rest of the subjects fell within the normal range (Table 1). None of the RA subjects displayed any asthma symptoms however and there was no clear relationship between those RA subjects who had positive methacholine tests and the other asthma related parameters we examined.

#### FEV_1_ and percentage reversibility

None of the RA group or the control subjects demonstrated greater than 15% bronchodilatability assessed by FEV1 after beta agonist administration. In contrast, 5 out of 15 asthma subjects demonstrated greater than 15% reversibility with the mean degree of reversibility being close to 15% for the CA group as a whole (Table 1), which was significantly greater than that of the RA subjects. There was no difference in reversibility between the RA and control groups.

#### Serum IgE and skin test positivity

Asthmatics (CA) had a significantly greater serum IgE than either RA or control groups. It was an objective, of our recruiting, to try and match RA and asthma subjects as far as possible for similar levels of “atopy” as defined by the number of positive skin tests to common aeroallergens. At a broad level, (atopic versus non atopic) our matching was good; however, there was a small but significant (p < 0.05) difference between the numbers of positive skin tests in the RA group and the CA group (Table 1).

### Sputum analysis

#### Sputum cell analysis

The cells within the sputum can provide an important reflection of the local inflammatory process within the airways in asthma. The total sputum cellularity was not significantly different between subject groups (data not shown)**.** The most striking and significant difference, between the subject groups, was in the eosinophil compartment. The CA group had >30 fold more eosinophils than the RA group (p < 0.01) (Table 2), while there was little evidence of differences in the proportions of neutrophils, macrophages, lymphocytes or bronchial epithelial cells between the subject groups (Table 2). It is notable that the sputum eosinophilia in the CA group occurred in the absence of any significant blood eosinophilia (Table 1).

**Table 2:** Total and differential counts in sputum

	**Control**	**CA**	**RA**
Total Cell Count (×10^6^)	9.56±8.67	3.38±2.32	6.60±5.93
% Eosinophils	0.043±0.012	7.06±11.19	0.292±0.439
% Neutrophils	37.16±21.56	22.64±14.24	29.96±22.25
% Macrophages	68.53±22.05	65.8±19.52	61.3±21.9
% Lymphocytes	0.98±0.92	1.72±1.40	0.98±0.87

#### Sputum fibrinogen and eosinophil cationic protein (ECP)

**Figure 2:**
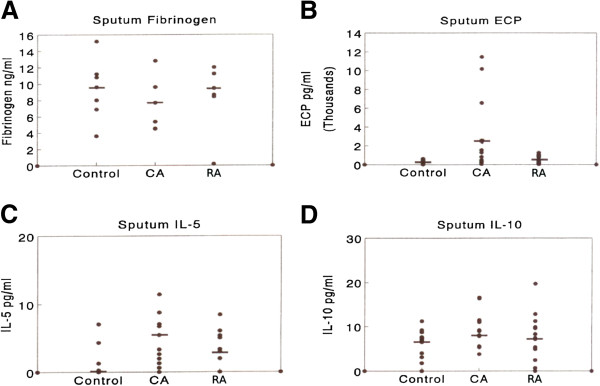
**Sputum mediator measurements from matched triplets of RA, CA and control group subjects (A) sputum fibrinogen, (B) sputum ECP, (C) sputum IL-5 (D) sputum IL-10.** Each point represents individual values or multiple identical values. Median values are denoted by bars. Significant (p < 0.05) differences in mediator levels between RA and CA groups were observed for sputum ECP and sputum IL-5 but not for sputum fibrinogen or sputum IL-10.

Increased levels of sputum fibrinogen have been widely used as a marker for airways damage and inflammation. In this study, we observed no significant differences between our three subject groups in terms of sputum fibrinogen level (Figure 2A). There was a significant (p < 0.05) increase in ECP in the CA group compared with the RA group. The RA group had levels of ECP very similar to the control group (Figure 2B). These results are consistent with the numbers of eosinophils observed within the sputum samples.

#### Sputum IL-5 and IL-10

The cytokines IL-5 and IL-10 were selected for study in the sputum supernatant for several reasons, including the critical nature of these cytokines in regulating airways inflammation, and the previous experience of ourselves and others in measuring cytokines at this site. There was a significant elevation in sputum IL-5 for asthmatic subjects (CA) compared with control subjects (Figure 2C). The RA group had intermediate levels of this sputum cytokine compared with the other two groups. In contrast, there were no substantial differences in the levels of IL-10 between the subject groups (Figure 2D).

### Peripheral blood cytokines

Preliminary experiments using normal and asthmatic (CA) subjects (data not shown) defined the optimal timing and stimulus doses required for studies of PBMC cytokine expression. We chose to examine the production of a number of type 1, type 2 and pro-inflammatory cytokines without fractionation of the cells, in order to obtain a picture of the cytokine production capability of the mixed cell population found *in vivo.* Our results revealed a number of significant differences in cytokine expression between the three subject groups.

#### IL-12

**Figure 3:**
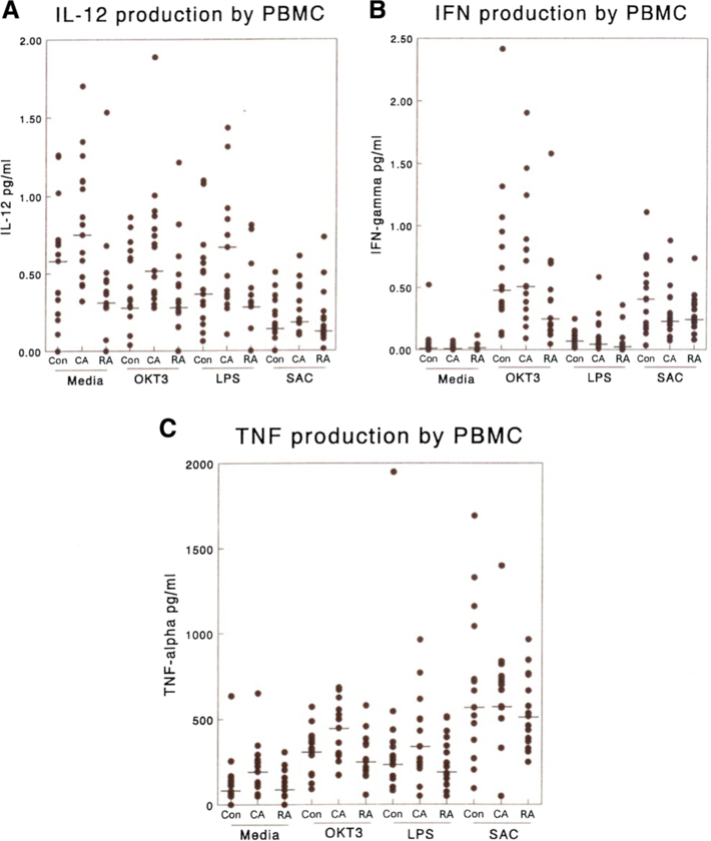
**The production of type 1 cytokines by PBMC from matched triplets of RA, CA and control subjects in response to a range of stimuli i.e. mouse anti-human CD3 (OKT3) 1 μg/ml, LPS 1 μg/ml and heat killed *S. aureus *Cowan strain 1 (0.01%w/v) (SAC). (A) IL-12 production at 72 h, (B) IFN-γ production at 24 h, (C) TNF-α production at 24 h.** A similar profile of results were obtained when IL-12 was assayed at 24 h (data not shown). All supernatant samples were assayed in parallel by ELISA. Each point denotes the result from individual supernatants or from multiple identical values. Bar denotes median value for the group. RA values were significantly lower than those in the CA group under the following conditions: For IL-12 (**A**) media alone, OKT3, LPS, For IFN-γ(**B**) OKT3, for TNF-α (**C**) Media alone OKT3, LPS.

Examination of IL-12 production at 72 h revealed that asthmatic subjects (CA) had consistently higher levels of IL-12 production than RA subjects or control subjects under a range of stimulation conditions (Figure 3A). None of the activating conditions elevated IL-12 production significantly under our culture conditions, in keeping with literature reports that single stimuli are not optimal for IL-12 induction. However, significant differences were observed between the groups when cells were incubated with media alone (p < 0.01) or activated with OKT3 (p < 0.05) or LPS (p < 0.05). Similar differences between groups were obtained when 1 L-12 production was examined at 24 h (data not shown) although the levels of IL-12 production were lower.

#### *IFN-*γ

IFN-γ is widely used as a marker for a Th-1 response in disease models. Examination of the production of IFN-γ in stimulated and control cultures after 24 hours revealed some important differences between our subject groups (Figure 3B). Both OKT3 and SAC were highly effective in inducing IFN-γ production in all subject groups. While there was no notable difference between asthmatic (CA) and control subjects in terms of IFN-γ production, RA subjects showed a striking decrease in IFN-γ production compared with either of these groups. RA subject PBMC production of IFN-γ was significantly less than the CA group when cells were incubated with media alone or with OKT3 (p < 0.05), but no such difference was observed when cells were activated with the less physiologically relevant A23187+ phorbol ester.

#### *TNF-*α

This cytokine is considered pivotal for the development and maintainance of an inflammatory response. Both lymphocytes and monocytes are recognised sources of TNF. OKT3, SAC, LPS and PdBu + A23187 were all effective stimuli for TNF-α production (Figure 3C). In general the CA group produced an elevated level of TNF-α compared with the control group. When compared with the RA subject group, the CA group PBMC produced significantly more TNF-α when cultured with media alone (p < 0.02) or OKT3 (p < 0.02) or LPS (p < 0.05). The RA subjects did not differ significantly from controls with respect to TNF-α production.

#### IL-4

**Figure 4:**
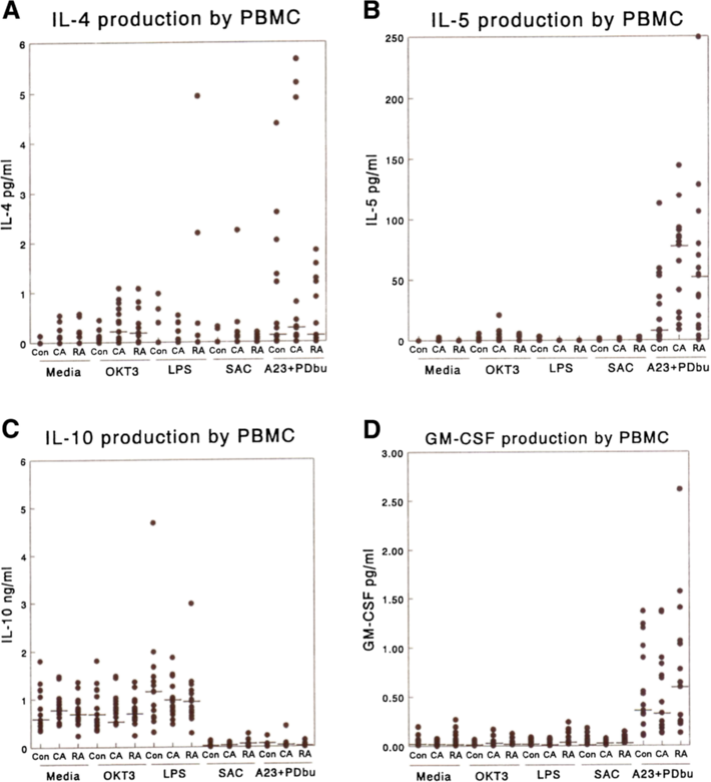
**The production of type 2 cytokines and GM-CSF by PBMC in response to a range of stimuli i.e. mouse anti-human CD3 (OKT3) 1 μg/ml, LPS 1 μg/ml and heat killed *S. aureus *Cowan strain 1 (0.01%w/v (SAC).** (**A**) IL-4 production at 24 h, (**B**) IL-5 production at 24 h, (**C**) IL-10 production at 24 h, (**D**) GM-CSF production at 24 h. No significant differences were noted between RA and CA groups for any of these cytokines under any of the activation conditions employed.

The levels of IL-4 produced in the PBMC cultures were very low and close to the limit of detection unless T-cells were specifically activated (mean limit of detection 0.13 pg/ml, Figure 4A). However, both the CA and RA groups, who were mainly atopic, produced significantly more IL-4 than control (non-atopic) subjects in the presence of OKT3. Since OKT3 is known to activate T-cells specifically this should reflect the activated T cell response in these subjects.

#### IL-5

Very low or undetectable levels of IL-5 were produced in response to most of the stimuli we employed at 24 h (Figure 4B) although a significant response was observed to OKT3 activation. There was a trend towards increased IL-5 production in the asthmatic subjects (CA) compared with the other subject groups, but it did not reach statistical significance. We did not observe, within the PBMC, the dramatic increases in IL-5 that were observed in the sputum of the CA subjects.

#### IL-10

Production of this cytokine was examined after 24 h and 72 h in culture with similar results. The data from 72 h cultures are illustrated in Figure 4C. There was a high level of endogenous IL-10 production in this culture system. The production of IL-10 was significantly enhanced in all groups of subjects by the addition of LPS and inhibited by the addition of SAC. There were no significant differences between CA and RA subjects under any of the stimulation conditions used at either time point.

#### GM-CSF

Low, but detectable levels of GM-CSF were produced by all subjects under most activation conditions (Figure 3D). None of the stimuli used, other than A23187 + PDBu, induced a significant enhancement of GM-CSF production. RA subjects produced significantly (p < 0.02) more GM-CSF than the CA subjects or controls following such activation.

### Flow cytometric analysis of PBMC

#### T cell subset, B cell and monocyte frequencies

**Figure 5:**
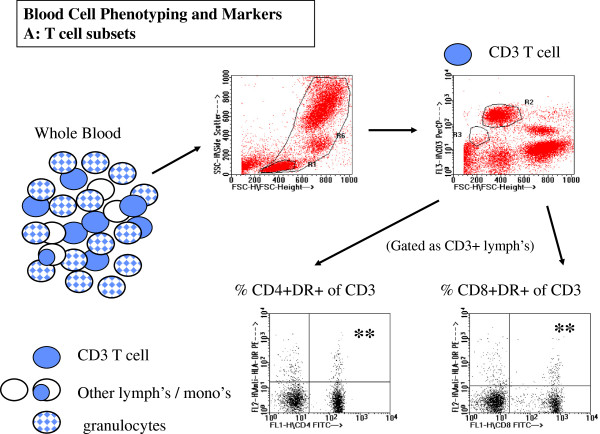
**Representative data for flow cytometry analysis of T lymphocyte subsets and their activation or differentiation markers.** CD3+ T lymphocytes (top right, R2 region) were first identified from whole blood cells defined by FSC and SCC (top left, R6 region). CD3+ T lymphocyte subsets were further defined by expression of CD4, CD8, and activation/differentiation markers such as HLA-DR (lower panels).

**Figure 6:**
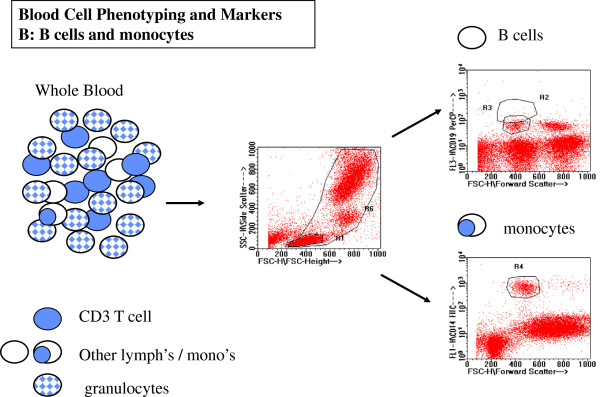
**Representative data for flow cytometry analysis of B lymphocytes and monocytes.** CD19+ B lymphocytes (top right R3 region) and CD14 monocytes (bottom right R4 region) are clearly distinguished from all other blood cells included in WBC gate for whole blood cells as indicated by R6 in the center plot.

**Figure 7:**
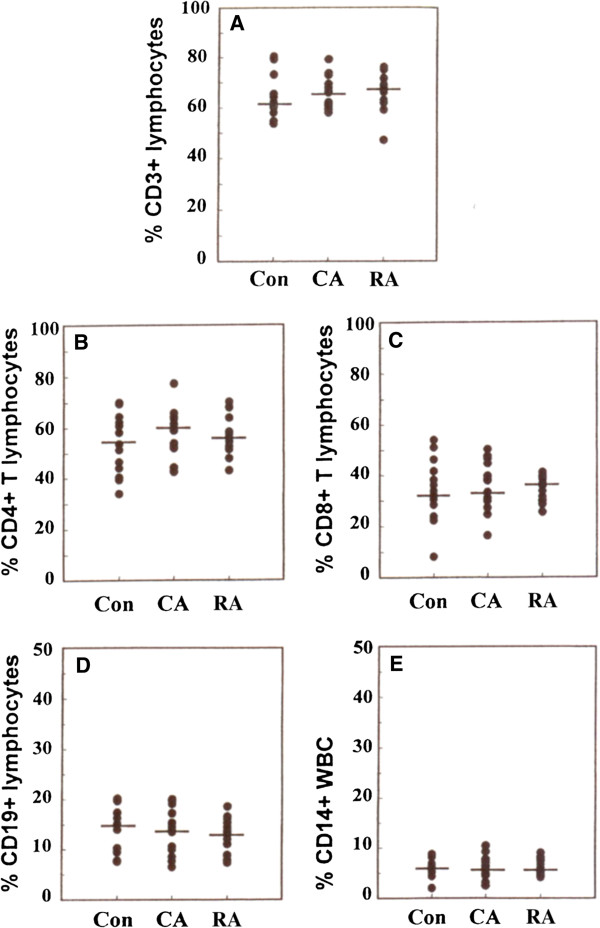
**Percentage of effector cells and their subsets present in blood from RA CA and control subjects, based on results of flow cytometric analysis of whole blood.** (**A**) percentage of lymphocytes expressing the CD3 T-cell marker. (**B**) the percentage of T-lymphocytes expressing CD4. (**C**) the percentage of T-lyphocytes expressing CD8. (**D**) the percentage of lymphocytes expressing the B-cell marker CD19. (**E**) the percentage of white blood cells expressing CD14. No significant differences were noted between the subject groups from any of these markers.

A multicolour analysis of T cell subsets, B cells and monocytes, was performed by flow cytometry of whole blood. The identification and analyses of various subsets and markers are illustrated in Figures 5 and 6. There were no significant differences between the three groups of subjects for the percentage of CD3+ (total T-cells), percentage of CD4+ or CD8+ T-cells or the percentage of B-cells among the lymphocyte population. There were also no differences in the percentage of monocytes among the total leukocytes (Figure 7).

#### Activated and memory T-cell subsets

**Figure 8:**
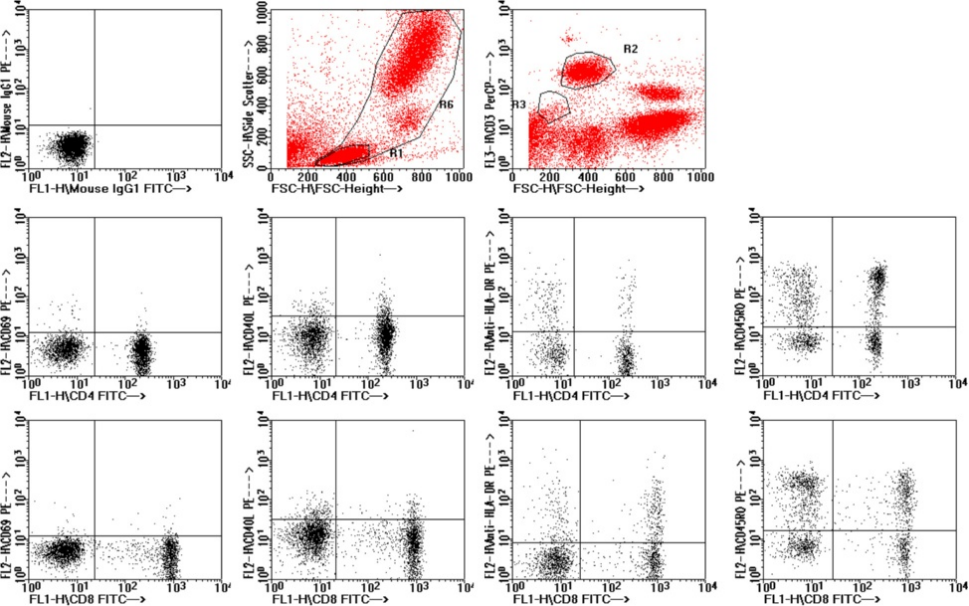
**Representative data illustrating analysis of T cell subsets and expression of activation and differentiation markers CD69, CD40L, HLA-DR, CD45RO.** Data are derived from normal control subject, illustrating typical frequency of cells expressing of these markers.

**Figure 9:**
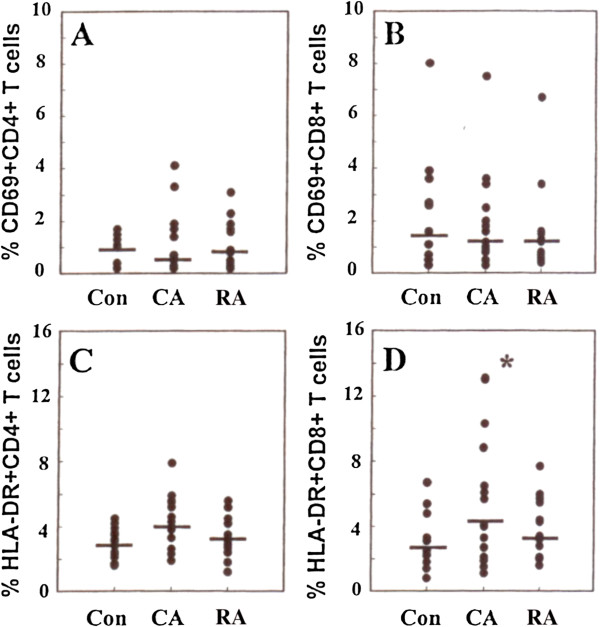
**The expression of activation markers on CD4 and CD8 T-cell subsets in blood from RA CA and control groups of subjects.** (**A**) percentage of CD69 positive CD4 positive T-cells. (**B**) percentage of CD69 positive CD8 positive T-cells. (**C**) percentage of HLA-DR positive CD4 positive T-cells (**D**) percentage of HLA-DR positive CD8 positive T-cells. Note significant (P < 0.05) increase in HLA-DR positive CD8 positive T-cells in CA group compared with control group.

**Figure 10:**
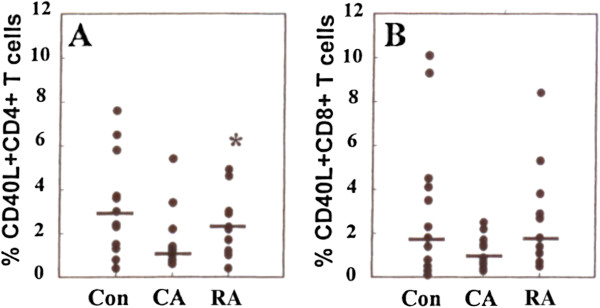
**CD40 ligand (CD40L) expression by T-cell subsets from the blood of RA, CA and control subjects.** (**A**) Percentage of CD40 L positive CD4 positive T-cells (**B**) percentage of CD40L positive CD8 positive T-cells. Note significant (P < 0.05) increase in CD40L positive CD4 positive T-cells in the RA group compared with the CA group.

Representative flow cytometric data showing typical analysis of T cell subset expression of activation markers CD69 and HLA-DR and differentiation markers CD40L and CD45RO are shown in Figure 8. CD69 expression indicates very recent activation of T-cells. The frequency of T cells expressing CD69 is normally very low among blood cells and many samples had no significant CD69 staining above background (0.5%) (Figures 9A and 9B). In contrast, examination of HLA-DR expression on T-cells, which is used as a marker of “near recent” activation, (typically among effector memory T cells) revealed an increase in HLA-DR expression of CD8 positive T-cells from the CA group compared with the RA group (p < 0.05) (Figure 9D). A similar trend was observed in the CD4 subset although statistical significance was not reached (Figure 9C). Examination of the expression of CD40 ligand by CD4+ T-cells also revealed differences between the subject groups with a decreased frequency of CD40 ligand expressing CD4+ cells in the CA group compared with the RA group (p < 0.05) (Figure 10A). A similar trend was observed in the CD8+ subset but without statistical significance.

CD45RO was used as a marker for memory/activated T-cells. CD45RO cells are generally phenotypically reciprocal to CD45RA (naive) T-cells with a low percentage of cells being positive for both markers. Neither CD45RO nor CD45RA expression on CD4 or CD8 subsets were different between the three subject groups (data not shown).

#### B-cell and monocyte activation markers

The frequency of B-cells expressing CD40, CD23 and HLA DR as well as the relative amounts (median fluorescence intensity) of these molecules were determined. All three markers are expressed by nearly 100% of B-cells in blood. In addition, the levels of expression of each of these markers can increase on activated B-cells. Circulating monocytes rarely express CD40 or CD23 but almost all express HLA-DR. However, CD40 and CD23 can be expressed *de novo* on monocytes following activation and HLA-DR can be up-regulated following appropriate stimulation of monocytes. There were no significant differences between the subject groups for the percentages of B-cells expressing each of these markers and no differences in the level of expression for CD40, CD23 or HLA-DR (Table 3). Similarly there was no difference in the level of monocyte expression of HLA-DR.

**Table 3:** Analysis of peripheral blood B cells, monocytes and their expression of CD40, CD23 and HLA-DR

**Cell Marker:**	**Group**	**Mean% (S.D.)**	Ave.MdFI* (S.D.)
B cells-CD23	Control	82.6 (9.4)	83.0 (39.5)
	CA	82.4(8.2)	85.6 (28.9)
	RA	79.6 (10.1)	90.6 (46.7)
B cells-CD40	Control	100	50.6 (18.6)
	CA	100	51.4 (15.2)
	RA	1000	53.4 (14.4)
B cells-HLA-DR	Control	100	975 (338)
	CA	100	1035 (277)
	RA	100	990 (340)
Monocytes-HLA-DR	Control	83.9 (12.6)	290 (118)
	CA	87.5 (12.6)	322 (120)
	RA	88.1 (9.2)	286 (124)

•MdFI = median fluorescence intensity.

## Discussion

The studies we describe here provide novel insights into the differences between children whose asthma has resolved spontaneously and those in which it remains symptomatic. Increased levels of sputum eosinophils and sputum IL-5, increased peripheral blood mononuclear cell-derived TNF-α, IL-12, and decreased IFN-γ levels were associated with on-going asthma symptoms and airway hyperresponsiveness. We did not demonstrate any clear differences in T or B cell subsets, memory cells or activation markers between children with ongoing asthma symptoms and those in whom symptoms had completely resolved. However, where subtle differences were noted between the CA and RA groups, the RA group reflected values closer to those of the control subjects.

Initial clinical examination revealed that many of the asymptomatic subjects remained hyperresponsive as defined by methacholine challenge tests. In a sample of 551 adolescent and young adult subjects Kolnaar et al. [34] observed that 42% of subjects exhibited airways hyper responsiveness, of which 70% were asymptomatic. These data would suggest a slightly lower rate in the population than our observation of hyper responsiveness in 8 of 15 asymptomatic RA subjects. A much larger study would be required to determine whether the rate of asymptomatic AHR in RA subjects was increased relative to the levels in the age matched general population. Other studies in childhood [35] have suggested that asymptomatic bronchial hyperresponsiveness, in this case observed after allergen challenge, can predict the development of asthma. The lack of AHR in many of the RA subjects suggests that the AHR resolves in many subjects in which asthma symptoms resolve. It is interesting that there was no evidence of an increase in FEV1 reversibility in the RA group compared with the control group despite the high rate of AHR.

Analysis of induced sputum provides a unique window on the inflammatory status of the airways of the RA subjects and their CA counterparts. The most striking difference observed between these groups was in the levels of eosinophils and ECP observed. The high levels of these markers observed in CA were expected, based on extensive literature reports of eosinophilic airways inflammation associated with symptomatic asthma. Notably, despite the presence of positive skin tests and AHR in over half the RA group, both the ECP and sputum eosinophil levels were comparable with those of non-atopic control subjects and significantly lower than those in the CA group. The increase in eosinophils was highly specific, as observed in other asthmatic populations [33,36] since no alterations were noted in the percentages of other cell types in the sputum. The local eosinophilia in the airways did not extend to significant changes in peripheral blood eosinophil numbers, although a peripheral eosinophilia has been described in some groups of asthmatics, especially in childhood [37,38].

The levels of cytokines in the sputum supernatants reflected the eosinophilic nature of the ongoing inflammatory process associated with disease. The IL-5 levels were significantly elevated in the CA group compared with the control group. In contrast, the predominately anti-inflammatory cytokine IL-10 was found in similar amounts in all subject groups. This finding does not support the concept that subjects in which asthma has resolved have enhanced local production of IL-10 compared with those in which the disease persists. The intermediate levels of IL-5 and TNF-α observed in RA subjects suggest that in some individuals elevated local concentrations of these cytokines remain, although in the absence of substantial eosinophilia.

The relationship between eosinophilic inflammation and AHR in asthma is not entirely clear. In murine models of asthma, some have shown that AHR and eosinophilic inflammation can be dissociated [39-41] while others have demonstrated a link between them via IL-5 [42-45]. Among the RA subjects, of whom 8 of 15 had AHR in the absence of symptoms, none had substantial levels of eosinophils in sputum, which seems to indicate that AHR can be present in certain subjects without a high level of eosinophilic inflammation. However, several of the RA subjects had increased IL-5 in their sputum in the absence of substantial eosinophilia, suggesting that perhaps this residual IL-5 may be associated with the residual AHR.

Our observations of elevated type 1 cytokine production by PBMC from CA subjects compared with control subjects were surprising in the context of a disease which is traditionally associated with inflammation driven by type 2 cytokines. There is recent information that supports the role of IFN-γ in normalizing asthma symptoms in childen [46]. However, several pieces of information from the existing literature support the concept of an important role for the type 1 cytokine cascade in symptomatic asthma. In early studies [47], TNF-α was observed as increased in the BAL fluid of subjects with “symptomatic” rather than quiescent disease. Elevated production of TNF-α and IFN-γ by bronchoalveolar leukocytes from a series of 11 patients with allergic asthma compared with control subjects [48]. Indeed, anti-TNF treatment has been reported to be beneficial in a subset of patients with severe asthma [49]. More recently, using whole blood cultures Magnan et al. [50] observed an overproduction of IFN-γ by CD8+ T-cells in the blood of asthmatic subjects which was related to asthma severity and suggested a role for IL-12 in inducing this response. Observations that IFN-γ inducing factor (IL-18) is increased in the airways of patients with atopic asthma support the concept that enhancing the type 1 cytokine cascade can be associated with the development of airways eosinophilia [51]. Interestingly an elevated type-1 cytokine response, in atopic subjects, has also been implicated in studies of atopic dermatitis [52]. Additional recent studies in human subjects and mice have reported the positive contribution of TH1 lymphocytes and IFN-γ to allergic lung inflammation [53-55]. It is also been reported that eosinophil-derived IFN-γ contribute to the persistence of airway hyperresponsivness and structural changes [56]. Differences in type 1 cytokine production between asthmatic and control subjects might be missed experimentally if either the subjects were not withdrawn from corticosteroid therapy appropriately or complex stimuli such as whole bacteria were employed. Our data suggest that results obtained using SAC as a cytokine-inducing stimulus for PBMC does not reflect the baseline levels of cytokine expression or the response to more direct stimulation of the T-cell or Toll-like receptor systems.

The CA and RA subjects did not differ substantially in their production of any of the type 2 cytokines we measured in PBMCs. Other RA and CA groups in this study were predominately atopic and several studies have suggested that enhanced ability to produce these cytokines reflects atopic status [57,58] rather than asthma specifically. Under appropriate stimulation conditions both IL-4 and IL-5 levels were elevated in supernatants from the CA and RA PBMC compared with the non atopic control group PBMC. This is in contrast with both IL-6 (data not shown) and IL-10 which were unchanged between groups regardless of the stimuli employed.

Flow cytometric studies of peripheral blood cells from all of the donors revealed few consistent or significant differences. All of the groups had similar proportions of T-cell subtypes, B-cells and monocytes suggesting that the changes we observed in cytokine expression were unlikely to be due to major alterations in cell distribution. The observation of elevated HLA-DR expression on the CD8 + ve T-cells from asthmatic subjects is consistent with the concept that these cells may be an important effector cell population or a source of type 1 cytokines in asthma. The increased expression of CD40 ligand that we observed in the asthma group compared with the control group could also relate to the presence of elevated type 1 cytokines since IL-12 has been demonstrated to be produced as a result of enhanced CD40/CD40L interactions [59].

This study is a unique attempt to evaluate the differences between carefully matched atopic subjects in whom asthma symptoms have or have not resolved. Previous studies of the immune parameters associated with growing out of asthma have concluded that lower serum IgE or fewer positive skin tests, male gender as well as less severe disease increase the likelihood that asthma will resolve spontaneously [9,10,20]. There has been some discussion that the resolution of asthma in late childhood/adolescence is temporary [60], although good prospective studies have not been performed in this area. The RA subjects examined by us have been surveyed 14–18 months after the completion of the study, and none of the subjects contacted had developed symptomatic asthma over this time period. Strategies aimed at the IL-5 dependent eosinophil recruitment into the airways or TNF-dependent airway hyperresponsiveness may hasten the resolution of asthma symptoms. Long-term prospective studies of children are necessary to investigate these strategies.

In conclusion, our studies of subjects who have “grown out” of asthma symptoms compared with those in whom disease continued has revealed that while many of these subjects retain some degree of AHR, there is little or no associated airways eosinophilia. As a group, those with continuous asthma demonstrated elevated production of type 1 cytokines compared with RA subjects. It is well recognised that corticosteroids are potent inhibitors of type 1 cytokine production, although this inhibition is temporary. The ongoing challenge is to develop new approaches to therapy which could induce the changes which take place during spontaneous disease remission. Further studies of subject groups such as the RA group we describe could provide important clues to this critical process.

## Competing interests

Dr. Nair is listed on a patent for a sputum filtration device and provides scientific advice to Cellometrics Inc, a university spin off company.

## Authors’ information

This study was supported by an unrestricted educational grant from AstraZeneca Canada. Dr Nair was supported by a Clinician Scientist Award from the Canadian Institutes of Health Research.
